# Function and Mechanism of Novel Histone Posttranslational Modifications in Health and Disease

**DOI:** 10.1155/2021/6635225

**Published:** 2021-03-03

**Authors:** Huiwen Xu, Maoyan Wu, Xiumei Ma, Wei Huang, Yong Xu

**Affiliations:** ^1^Department of Endocrinology and Metabolism, The Affiliated Hospital of Southwest Medical University, Luzhou, Sichuan, China 646000; ^2^Sichuan Clinical Research Center for Nephropathy, Luzhou, Sichuan, China 646000; ^3^Cardiovascular and Metabolic Diseases Key Laboratory of Luzhou, Luzhou, Sichuan, China 646000; ^4^Faculty of Chinese Medicine, Macau University of Science and Technology, Avenida Wai Long, Taipa, Macau, China

## Abstract

Histone posttranslational modifications (HPTMs) are crucial epigenetic mechanisms regulating various biological events. Different types of HPTMs characterize and shape functional chromatin states alone or in combination, and dedicated effector proteins selectively recognize these modifications for gene expression. The dysregulation of HPTM recognition events takes part in human diseases. With the application of mass spectrometry- (MS-) based proteomics, novel histone lysine acylation has been successively discovered, e.g., propionylation, butyrylation, 2-hydroxyisobutyrylation, *β*-hydroxybutyrylation, malonylation, succinylation, crotonylation, glutarylation, and lactylation. These nine types of modifications expand the repertoire of HPTMs and regulate chromatin remodeling, gene expression, cell cycle, and cellular metabolism. Recent researches show that HPTMs have a close connection with the pathogenesis of cancer, metabolic diseases, neuropsychiatric disorders, infertility, kidney diseases, and acquired immunodeficiency syndrome (AIDS). This review focuses on the chemical structure, sites, functions of these novel HPTMs, and underlying mechanism in gene expression, providing a glimpse into their complex regulation in health and disease.

## 1. Introduction

**Figure 1:**
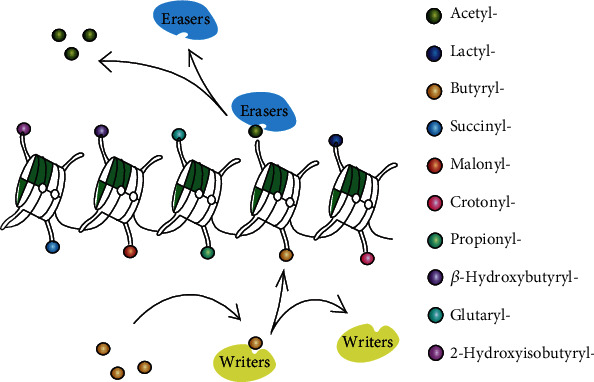
A dynamic process of HPTMs. Histone octamers (H2A, H2B, H3, and H4) wrapped around with the double helix make up the core construction of the nucleosome. The addition and removal of HPTMs usually take place on the N-terminal tails of histone catalyzed by specific enzymes called “writers” and “erasers.” The “writers” are enzyme/enzyme complexes that catalyze the covalent modifications of specific residues, and “erasers” are specific enzyme/enzyme complexes that remove the modifications. HPTMs include acetylation, propionylation, butyrylation, 2-hydroxyisobutyrylation, *β*-hydroxybutyrylation, succinylation, malonylation, glutarylation, crotonylation, and lactylation, which can happen alone or in combination to affect gene expression.

Epigenetics includes, among others, histone posttranslational modifications (HPTMs), DNA methylation, and RNA interference. HPTMs can change the conformation of histone and affect chromatin-associated nuclear processes to control development, tissue differentiation, cellular responsiveness, and biological phenotype without altering the underlying genetic sequence. Histone acylations are less known epigenetic markers in comparison to other PTHMs, showing a reversible, dynamic, and conserved character. The addition and removal of histone acylations are modulated by specific enzymes or enzyme complexes called “writers” and “erasers,” forming transcriptional signals read by effector proteins called “readers” to affect downstream signal pathways and promote various biological events [1] (shown in Figure 1). HPTMs are affected by the external environment and metabolic states and can regulate gene activity or gene silencing involved in inflammation [2, 3], cancer [4, 5], cardiovascular diseases [6], kidney diseases [7, 8], metabolic diseases [9, 10], and neuropsychiatric disorders [11, 12].

Epigenetic changes play a causal role in diseases and occur long before diseases develop. Histone lysine acetylation (Kac) is a classical histone acylation that has an effect related to DNA damage and repair, cell-cycle control, chromatin architecture, RNA splicing, and transcription [13]. Methylation takes part in gene repression or activation, depending on the sites and degree [14, 15]. Phosphorylation is a common modification associated with activation of transcription, apoptosis, and repair of DNA damage [16, 17]. Likewise, ubiquitination and SUMOylation have multiple nuclear functions, including transcription, mRNA processing, DNA replication, and DNA-damage response [18, 19]. Recently, a breakthrough in high-resolution mass spectrometry- (MS-) based proteomics has found that the side chain of the histone lysine can be modified by nine novel types of histone lysine (K) acylations: propionylation (pr) [20], butyrylation (bu) [20], crotonylation (cr) [21], malonylation (ma) [22], succinylation (succ) [22], glutarylation (glu) [23], 2-hydroxyisobutyrylation (hib) [24], *β*-hydroxybutyrylation (bhb) [25], and lactylation (la) [26]. Emerging evidence has suggested that HPTMs are strongly associated with the pathogenesis of cancer, neuropsychiatric disorders, nephropathy, infertility, metabolic disease, and even the latency of Acquired Immune Deficiency Syndrome (AIDS). Histone acylations have emerged as hot topics in the field, given their intrinsic role in bridging cellular metabolism and epigenetic regulation. For instance, Kac is often associated with chronic inflammatory conditions and cancer; however, other types of nonacetyl acylation may compete and replace Kac in some sites, resulting in anti-inflammatory, antitumor, or unknown effects. This review focuses on the chemical structure, sites, functions of these novel HPTMs, and underlying mechanism in gene expression, providing a glimpse into their complex regulation in health and disease.

## 2. The Specific Donor, Enzymes, and Process of Novel HPTMs

The levels of diverse histone lysine acylations represent a snapshot of cellular metabolism and conditions. In recent years, scholars have proposed various models to explain how HPTMs are regulated by the metabolism of various acyl-CoA and how they modulate gene expression. The occurrence of several metabolite-sensitive acyls has clarified the intricate link between histone lysine acylation and cellular metabolism. Acyl-coenzyme A (acyl-CoA) [27], as the specific donor of the acyl group, is a metabolic intermediate in many metabolic pathways. For example, cellular glycolipid metabolism can produce some endogenous acyl-CoAs (e.g., Ac-CoA, Hib-CoA, Succ-CoA, Bhb-CoA, and Ma-CoA). Some specific acyl-CoAs (i.e., Bu-CoA, Pr-CoA, and Cr-CoA) are generated depending on the supply of corresponding short- or long-chain fatty acids (SCFAs or LCFAs), which are derived from anaerobic bacteria fermentation in the intestine [28]. There is a study that has proved that treating cells with either isotopic or radio-labeled SCFAs leads to the labeling of histone proteins by heavy or radioactive acyl, which suggests that fatty acids can transform into their cognate acyl-CoAs that are then used directly as cofactors in the histone acylation reactions [29]. The competition of different acyl-CoAs establishes the different acylation states of histone. Acyl-CoA synthetase short-chain family member 2 (ACSS2), also known as acyl-CoA synthetase (ACS), is a key enzyme that can form complexes with transcription activators at the promoter regions to support histone acylation and activate gene expression programs [30]. ACSS2 can catalyze the generation of acyl-CoA and maintain the level of acyl-CoA.

There are specific enzymes and enzyme complexes for the addition and removal of HPTMs, correspondingly called “writers” and “erasers.” Histone acetyltransferases (HATs) and histone deacetylases (HDACs) are canonical enzymes of histone Kac [13], which are also involved in nonacetyl acylation. HATs are divided into three significant families by their sequence and structural features, such as Gcn5-related N-acetyltransferases (GNATs), the P300/CREB-binding protein (P300/CBP), and the MYST family (Moz, Ybf2, Sas2, and Tip60). HDAC family members are mainly classified into two categories: Zn^2+^-dependent HDACs (class I, II, and IV enzymes composed by HDAC1–11) and NAD^+^-dependent HDACs (class III enzymes formed by sirtuin proteins 1–7). Class I enzymes (HDAC1–3, 8) mainly localize to the nucleus, while class II (HDAC4–7, 9–10) and class IV (HDAC11) enzymes generally shuttle between the nucleus and cytoplasm.

## 3. Classification of Novel HPTM Repertoire

**Figure 2:**
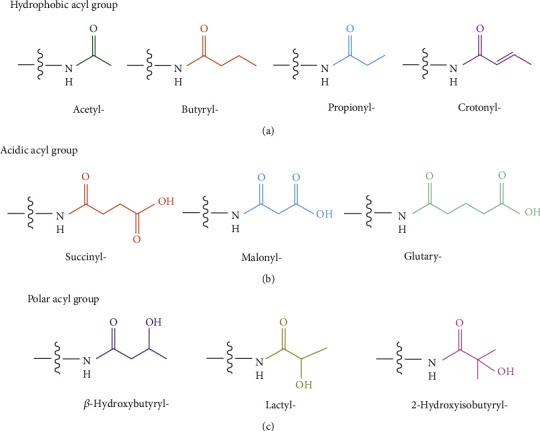
The structure and classification of acyl groups. Acyls are divided into three groups according to their chemical structure: (a) hydrophobic acyl group, including acetyl, propionyl, butyryl, and crotonyl modifications, extends hydrocarbon chains that increase the hydrophobicity and bulk of the Lys residue; (b) acidic acyl group, including succinyl, malonyl, and glutaryl modifications, changes the charge into the negative; (c) polar acyl group, including *β*-hydroxybutyryl, 2-hydroxyisobutyryl, and lactyl modifications, enables the modified Lys to form hydrogen bonds with other molecules.

**Figure 3:**
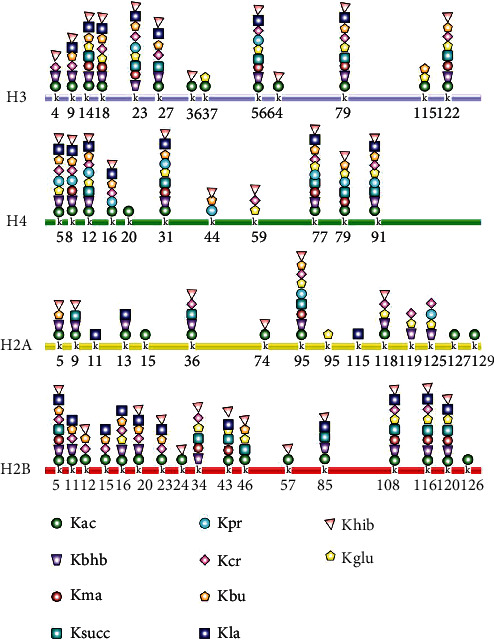
The acylation sites on HPTMs. Novel HPTM sites identified on the four core histones (H3, H4, H2A, and H2B) mostly overlap with known histone acylation sites. Some of them display a Kac-nonoverlapping modification pattern with a unique consequence for different epigenetic phenomena. For example, Kbu competitively inhibits Kac on H4K5 in spermatogenesis by restricting acetyl groups binding to the bromodomains. Kac: lysine acetylation; Kbhb: lysine *β*-hydroxybutyrylation; Kma: lysine malonylation; Ksucc: lysine succinylation; Kpr: lysine propionylation; Kcr: lysine crotonylation; Kbu: lysine butyrylation; Kla: lysine lactylation; Khib: lysine 2-hydroxyisobutyrylation; Kglu: lysine glutarylation.

The core nucleosome is a basic unit of chromatin and consists of four pairs of histones (H2A, H2B, H3, and H4) with the double helix wrapped around. For lysine residues in histone, the positively charged *ε*-amine can be positioned either within catalytic sites or more often on the solvent-exposed protein surface. The lone-pair electrons of the *ε*-amine of lysine, combined with its preferred localization on the protein surface regions, make lysine accessible to diverse modifications [15]. These nine findings of HPTMs are distinct from histone Kac in their hydrocarbon chain lengths, hydrophobicity, and charge, dramatically expanding the repertoire of HPTM. According to the chemical construction of the acyl group, HPTMs can be divided into three types (see in Figure 2). First, the hydrophobic acyl groups (e.g., propionyl, butyryl, and crotonyl) neutralize the positive charge of lysine residues. Second, the acidic acyl groups (malonyl, succinyl, and glutaryl) convert the positive charge of lysine residues into a negative charge. Third, the hydroxylated acyl groups (*β*-hydroxybutyryl, 2-hydroxyisobutyryl, and lactyl) allow the formation of hydrogen bonds with other molecules [31]. Notably, most of their acylation sites (see in Figure 3) overlap with the locations of Kac, showing a competitive or synergistic effect. Some display nonoverlapping modification patterns, which may elicit different consequences for epigenetic phenomena. This section outlines the writers, erasers, and readers of these novel HPTMs and summarizes the biological effects (summarized in Table 1).

### 3.1. Propionylation and Butyrylation

Histone Kpr and Kbu are detected by labeling and peptide mapping in vivo and in vitro [20]. Both show highly conserved properties in eukaryotes, such as *yeast* cells, mouse livers, and leukemia cell line U937 [32–34]. Propionyl-CoA and butyryl-CoA are the corresponding substrates donating propionyl and butyryl, which are essential to the acylation reaction of Kpr and Kbu. Catalyzed by acyltransferases to form hydrophobic acyl groups, Kpr and Kbu extend hydrocarbon chains to increase the hydrophobicity and the bulk of the lysine residue beyond that of Kac [31]. P300/CBP is the common acyltransferase and can rapidly promote Kpr and Kbu [33, 35]. GCN5 and MYSTs are the specific writers of Kpr but remain unclear in Kbu. SIRT1–3 can remove the propionyl and butyryl group from histone lysine [36]. Kpr and Kbu are highly dynamic and preferentially enriched in the promoters of active genes and recognized by acylation-state-specific reader proteins, such as bromodomain and PHD finger-containing protein 1 (BRPF1) [37].

These two modifications may promote a higher transcriptional output. A study has discovered that Kpr plays an essential role in regulating metabolism because it mostly occurs in proteins associated with energy production and conversion [38]. Kbu has a similar effect, although its abundance in healthy cells is quite low. In addition to directly stimulating transcription, butyrylation can competitively inhibit acetylation on H4K5 in spermatogenesis by restricting the acetyl group binding to the bromodomains of bromodomain testis-specific (BRDT), a testis-specific protein of unknown function containing two bromodomains. It will impact the final male epigenome features [39].

### 3.2. Malonylation, Succinylation, and Glutarylation

Histone Kma was initially found in *E. coli* [40] and HeLa S3 cells [41] and later widely found in mammalian cell lines, liver tissues, and nonmammalian species. Malonyl-CoA, as the substrate in the reaction, can regulate the malonylation level via acyltransferase catalysis [42]. SIRT5 can catalyze lysine demalonylation both in vitro and in vivo [40]. SIRT2 performs demalonylation in yeast [43]. Malonylation in proteins is always related to cellular metabolism, e.g., glycolysis pathway, glucose, and fatty acid metabolism. Even though several groups have demonstrated the effect of histone Kma in metabolism regulation [10, 44], the precise mechanism of histone Kma is still unclear.

Ksucc is a common HPTM that has been detected in *E. coli*, *yeast* cells, *Toxoplasma gondii*, HeLa cells, and mouse liver [22, 45]. By labeling HeLa cells with isotopic succinate, Xie et al. [22] identified Ksucc in histone peptides and demonstrated that it was regulated by the concentration of succinyl-CoA. Studies have suggested that succinyl can bind to P300/CBP or GCN5 and be recognized by the YEATS domain-like Glioma-Amplified Sequence-41 (GAS41) [46, 47]. Succinylation is more apt to occur on mitochondrial proteins in some eukaryotic organisms because succinyl-CoA is an essential intermediate metabolite at the central nodes of the tricarboxylic acid (TCA) cycle. The level of Ksucc has a close association with SIRT5 in mitochondria and SIRT7 [48]. Several studies have suggested that protein Ksucc is involved in energy metabolism, but the biological significance of Ksucc as a type of HPTM remains unknown.

Kglu was found on the core histones in HeLa cells and *Saccharomyces cerevisiae* as a new histone mark. It is rich in promoters of highly expressed genes and regulated by GCN5 and SIRT7 [23]. Similarly, glutaryl-CoA can affect the existence and abundance of the corresponding Kglu in different types and stages of cells and respond to various environmental stimuli. Current research has shown that Kglu is associated with cell regulation in response to DNA damage. Its identification raises new questions about the functions, mechanisms, and scope by which cellular metabolites regulate various signaling pathways.

### 3.3. *β*-Hydroxybutyrylation, 2-Hydroxyisobutyrylation, and Lactylation

Histone Kbhb is a high-profile mark present at high levels when *β*-hydroxybutyrate (BHB) concentrations are high. BHB provides an energy source to the heart and brain during starvation, a type of ketogenic diet. Regarded as a specific cofactor, *β*-hydroxybutyryl-CoA can catalyze the process of Kbhb in a concentration-dependent manner. Little is known about the writers and erasers for Kbhb. However, P300 can add *β*-hydroxybutyryl to histones, and SIRT3 can reverse this process [31]. Both class I and III HDACs have been reported to have histone de-*β*-hydroxybutyrate activity. Kbhb is mainly enriched on active gene promoters and associated with upregulated genes in starvation-responsive metabolic pathways [25]. BHB and Kbhb have been reported in the research of depression and cancer. However, the role and molecular mechanism of Kbhb are complicated and need further exploration.

Histone Khib is an evolutionarily conserved and dynamic mark, affecting active gene transcription and cellular proliferation. 2-Hydroxyisobutyrate (HIB), the isomer of *β*-hydroxybutyrate, is the substrate of Khib. According to Dai et al.'s report, histone Khib has a distinct genomic distribution from that of Kac and Kcr during male germ cell differentiation [24]. It is likely to be the result of an enzymatic reaction using the high-energy donor molecule, Hib-CoA. Experiments using recombinant HDACs and acid-extracted core histones in vitro suggest that HDAC1–3 can remove the 2-hydroxyisobutyryl group. However, this result needs to be confirmed in cells. Many other 2-hydroxyisobutyrylated proteins are involved in metabolic pathways (e.g., purine metabolism, pentose phosphate pathway, glycolysis, and gluconeogenesis). Whether these pathways are related to histone Khib is not clear.

Histone Kla is a new HPTM, first identified by Zhang et al. [26]. Lactate-derived lactylation of histone lysine residues has been observed in human and mouse cells. This epigenetic modification is catalyzed by P300 and directly stimulates gene transcription from chromatin. There is a potential connection with homeostatic genes, such as *Arg1*. This interesting finding may represent a new direction for investigating the signal pathways and mechanisms associated with Kla.

### 3.4. Crotonylation

Histone Kcr, which has been found in the genomes of both human somatic and mouse male germ cells, acts as a marker of active promoters and potential enhancers that regulate gene transcription [49]. Kcr is unique in histone acylation because crotonyl contains a four-carbon planar chemical moiety containing a C=C bond, which makes it possess some particular function [50]. Kcr is identified by readers, such as the YEATS domain, DPF domain, and CDYL [51]. It can be reversibly catalyzed by protein crotonyl-transferases and decrotonylases. Sabari et al. [52] determined that only P300 shows measurable histone crotonyl-transferase activity. The sirtuin family (e.g., SIRT1, SIRT2, and SIRT3) catalyzes the hydrolysis of lysine-crotonylated histone peptides and proteins [53, 54]. Overexpression of class I HDACs in HeLa cells can reduce the overall Kcr levels [55]. Histone Kcr is highly dynamic and may activate or repress transcription. It regulates the cell cycle and metabolism to a higher degree than histone Kac in a gene- or environment-dependent manner.

## 4. The Dysregulation of Novel HPTMs Take Part in the Pathophysiology of Diseases

As one of the epigenetic mechanisms, HPTMs play a dramatic role in some complex diseases by regulating the crucial functions of many eukaryotic proteins involved in cellular metabolism, cell differentiation, inflammation, apoptosis, aging, growth, angiogenesis, and cancer. Histone Kac regulates transcription by weakening the electrostatic interactions between histone and DNA. It provides a permissive chromatin environment for transcription, which activates cellular signaling pathways, such as the NF-*κ*B and SMAD pathways, by increasing cytokine expression to promote tissue inflammation, fibrosis, and acute or chronic injury. Although the mechanisms of novel HPTMs in different diseases are still unclear, there are already studies that have attempted to explore the connection between these modifications and disease (summarized in Table 2).

### 4.1. Novel HPTMs and Cancer

Increasing evidence suggests a critical role for epigenetic processes in cancer causation, progression, and treatment. Singh et al. [56] showed that HDAC inhibitors (HDIs), target enzymes in anticancer drug research, induce histone Kpr in addition to Kac, suggesting that the level of Kpr has a potential connection with tumorigenesis. Xu et al. [57] demonstrated that suberoylanilide hydroxamic acid (SAHA), a clinically approved HDI for cutaneous T-cell lymphoma, has promising clinical benefits against neuroblastoma. SAHA inhibits cell viability and also upregulates histone Kac (H3K9ac, H3K27ac) and Kbu (H2BK5bu, H4K12bu), causing proteome changes. The downregulated proteins are associated with multiple pathways (e.g., TCA cycle; the degradation of valine, leucine, and isoleucine; fatty acid metabolism; and all major glucose metabolism pathways), and the upregulated proteins are highly enriched in the cell-cycle and nucleotide-related processes, including DNA replication, spliceosome, and mismatch repair (e.g., RAD 17 and RAD 18). These regulated proteins may correlate directly with Kac- and Kbu-induced early gene expression profile changes or indirectly with other secondary effects, emphasizing the importance of epigenetic changes in mediating the loss of gene function in cancer. Zhang et al. [5] performed a comparative analysis of histone marks on a global level in two phenotypes of esophageal squamous cell carcinoma (ESCC) with different invasiveness (TE3, KYSE180) using an MS-based proteomics approach. They obtained comprehensive profiling of histone H3 and H4 acylation reactions, including Kbu (H3K18bu, H3K23bu, H3K79bu, and H4K77bu in the TE3 cell). Kbu is present in fairly low abundance compared to Kac at the same site and is associated with recurrence-free survival in different ESCC stages. It is reasonable to hypothesize that the imbalance between Kac and Kbu may be involved in ESCC. However, the role of Kbu in the pathogenesis of ESCC is still unclear. Fellows et al. [58] noticed that Kcr is hugely abundant in intestinal epithelial cells, especially in the crypt of the small intestine and colon. The peak of crotonylation on H3K18 has been found in many genes related to the cell cycle and cancer pathways (e.g., Hippo and cGMP-PKG signaling). It should be noted that the dysfunction of the Kcr reader, the YEATS domain, is often linked to human cancer [51]. Kcr is associated with hepatocellular carcinoma (HCC) TNM stage by inhibiting hepatoma cell migration and proliferation [59]. Besides, Kcr is downregulated in liver, stomach, and kidney carcinomas, but upregulated in thyroid, esophagus, colon, pancreas, and lung carcinomas.

There is an argument that ketone bodies will contribute to the stemness of cancer cells [60]. In contrast, Allen et al. [4] found that ketogenic diets can inhibit xenograft tumor growth potentially in human patients. These diets are under evaluation as adjunctive treatments for patients with brain tumors and other malignancies. Xie et al. [25] predicted that elevating histone Kbhb levels may induce a more tumor repressive gene expression pattern, which may work in part by altering the chromatin structure. Interestingly, Zhang et al. [26] discovered a new type of histone modification, Kla. Histone Kla has a close connection with lactate, a cellular metabolite in cancer, and is regulated dynamically by the amount of glycolysis available in cells. Increased Kla in the late phase of M1 macrophage polarization induces the homeostatic gene Arg1, perhaps to help repair collateral damage incurred by the host. The existence of histone Kla is likely to fill the knowledge gap in our understanding of diverse physiopathology (i.e., infection and cancer). It is encouraging that these novel HPTMs can modulate gene expression and may be new therapeutic targets of diseases.

### 4.2. Novel HPTMs and Metabolic Disease

Metabolic disease is a systemic low-grade inflammatory disease caused by both genetic and environmental factors. There is no dispute that epigenetics and HPTMs are involved in the occurrence and development of metabolic disorders. Kebede et al. [34] have proposed that histone Kpr, Kac, and Kbu may act in combination to promote high transcriptional output and couple cellular metabolism with chromatin structure and function. To explore whether novel HPTMs play an essential part in metabolic diseases, scholars have studied the prediabetic high-fat diet-induced obese (DIO) mouse model in vivo. They have found that Kpr, Kbu, and Kmal are downregulated in liver tissues [9]. Clinical data also confirm that malonyl-CoA levels are increased in the muscles of obese and type 2 diabetic subjects, suggesting that these markers may be associated with obesity and type 2 diabetes mellitus [61]. ChIP-seq and RNA-seq analyses have demonstrated that Kbhb in H3K9 is an active gene mark under conditions of starvation or STZ-induced diabetic ketosis and associated with genes upregulated in starvation-responsive metabolic pathways (e.g., amino acid catabolism, circadian rhythm, redox balance, PPAR signaling pathway, and insulin signaling pathway) [25]. There is a study providing evidence that the stimulation of ketogenesis following starvation leads to an increase in Kbhb and Kbu [62]. Similarly, Du et al. [10] observed that increased malonylated proteins in both ob/ob and db/db diabetic mouse models, particularly in liver tissue, affected glucose and fatty acid metabolic pathways and reversed insulin resistance. However, it is unclear whether these proteins could be locked in histone lysine. Zhang et al. presented a comprehensive map of histone Kma in the human fetal brain and found a significant elevation of lysine malonylation level in brain tissues from mice with diabetes-induced neural tube defects (NTDs) [44]. These studies offer additional lines of evidence on possible links between cellular metabolism and epigenetic regulation by new types of histone acylation in diabetes development. However, the specific regulatory effects and molecular mechanisms of these novel HPTMs in metabolic diseases need to be deeply explored.

### 4.3. Novel HPTMs and Neuropsychiatric Disease

The neuropsychiatric disease consists of abnormal neurological and mental conditions (e.g., neurodegeneration, epilepsy, and depression) manifested by cognitive and behavioral disorders and psychological changes. The causes of neuropsychiatric disease vary and are complicated, with no effective treatment. Previous studies showed that newly discovered HPTMs are closely related to the occurrence and development of these diseases. Chen [12] reported that exogenous BHB ameliorates depressive behaviors by upregulating Kbhb and activating the brain-derived neurotrophic factor (BDNF) gene in depressed mouse models. Kbhb may be the key to ketogenic diets, improving medically refractory epilepsy with a more extensive and significant neuroprotective effect than a restricted diet regimen [63]. There is also a report suggesting that Kbhb may be associated with depression accompanied by apparent metabolic disorders. In a recent study, CDYL-mediated histone Kcr was shown to regulate stress-induced depression. The level of Kcr in the medial prefrontal cortex is lower in chronic social defeat stress- (CSDS-) induced stress-susceptible rodents concurrently with selective upregulation of CDYL. These results provide a possible therapeutic orientation for major depressive disorder (MDD) [11].

### 4.4. Novel HPTMs and Reproductive Disease

Among the many factors of male sterility, spermatogenesis disorders can cause azoospermia and severe oligospermia. Several studies have shown that some novel HPTMs are associated with sperm maturation disorders and dysgenesis. These abnormalities are closely connected with the bromodomain of BRDT, which can recognize histone Kac and recruits transcription complexes from chromatin to promote specific gene expression [64]. Goudarzi et al. [39] reported that highly active Brdt-bound gene promoters systematically harbor competing Kac and Kbu marks in H4K5 and H4K8. Kbu also marks retarded histone removal during late spermatogenesis. Alternating Kac and Kbu on H4 may impact the ultimate male epigenomic features. Kcr is a specific mark of active sex chromosome-linked genes in postmeiotic male germ cells that are tightly correlated with a specific haploid cell gene expression program. It may be an essential signal controlling male germ cell differentiation [54]. Liu et al. [65] confirmed that CDYL downregulates the level of Kcr in vitro, leading to the maladjustment of Kcr in CDYL transgenic mice in vivo, decrease in epididymis sperm count and sperm cell activity, and hypofunction in male fertility. However, no studies have reported an association between HPTMS and oocyte or female infertility for now. Studies in this field need further exploration to improve our understanding of the relationship between novel HPTMs and reproductive disease.

### 4.5. Novel HPTMs and Kidney Disease

Kidney disease can be divided into acute kidney injury (AKI) and chronic kidney disease (CKD), part of which will gradually develop into end-stage renal disease (ESRD). Epigenetics, especially HPTMs, are involved in its pathogenesis. The tumor necrosis factor-like weak inducer of apoptosis (TWEAK) is a vital contributor to kidney injury. Ruiz-Andres et al. [7] found that histone Kcr is increased in TWEAK-stimulated tubular cells and tissues in an AKI model. Moreover, PGC-1*α* and SIRT3 are upregulated, and CCL-2 is downregulated. Further study confirmed that experimental AKI and renal function decline could be prevented by increasing crotonic acid levels. Therefore, we can speculate that Kcr may have a protective effect against AKI and facilitate renal function recovery. Chen et al. [66] measured the overall Kcr sites in the proteome of CKD and maintenance hemodialysis patients (MHP). They found that the level of Kcr is downregulated in hemodialysis patients but increased in CKD patients, suggesting an important regulatory role for Kcr and a dynamic change in its levels in the development of AKI and during the AKI-to-CKD transition. It will be important to elucidate the unique mechanism of Kcr in the proteome, particularly in histones.

Recently, Shimazu et al. provided us with some new ideas on how *β*-hydroxybutyrate is related to kidney disease. They found that *β*-hydroxybutyrate conferred substantial protection against oxidative stress in the mouse kidney by inhibiting HDAC activity and enhancing histone Kac at the Foxo3a and Mt2 promoters. However, its protective effect was weaker than the HDI [8]. In contrast, another study found that butyrate in endothelial cells suppresses gene expression and LPS-induced secretion of several proinflammatory genes. At the same time, *β*-hydroxybutyrate acted as a slightly proinflammatory molecule [67]. Additional experimental and clinical research is needed to clarify these conflicting results.

### 4.6. Novel HPTMs and Infectious Diseases

Infectious diseases are extremely complex biological processes closely related to autoimmune and inflammation. Microorganisms induce epigenetic modification during infection, such as repression or promotion of expression of different inflammasomes [68]. The epigenetic mechanisms also can be utilized by immune cells to regulate their gene transcription in response to infections [69]. Some reports have mentioned HPTMs, e.g., phosphorylation, acetylation, or methylation have played a critical role in the pathogenesis of infections, including viral infection (herpes simplex virus (HSV) [70], HBV [71], and HIV [72]), model of sepsis [73], and so on. However, there are few relevant mechanism studies about novel HPTMs and infectious bacterial or viral diseases yet.

Recently, a study focused on HIV infection and AIDS has mentioned that novel HPTMs play a role in the developing process. There is a time lag between infection with HIV and developing AIDS. Interestingly, viral latency is epigenetically regulated. ACSS2 induction is highly synergistic with vorinostat, an HDAC inhibitor, in reactivating latent HIV; however, the molecular mechanism is unknown. In Jiang et al.'s [74] report, histone Kcr is an epigenetic modification of the HIV long-terminal repeat that regulates HIV transcription and latency. Suppression of histone Kcr inhibits reactivation of latent HIV, and disruption of HIV latency was observed by histone Kcr following the induction of ACSS2 in vitro and ex vivo. In other words, inhibition of Kcr may support the maintenance of viral latency of AIDS, suggesting Kcr could be a target marker for controlling and reducing the morbidity of AIDS. These findings also raise interest in the potential mechanism and function of novel HPTMs.

## 5. Conclusions and Prospects

**Figure 4:**
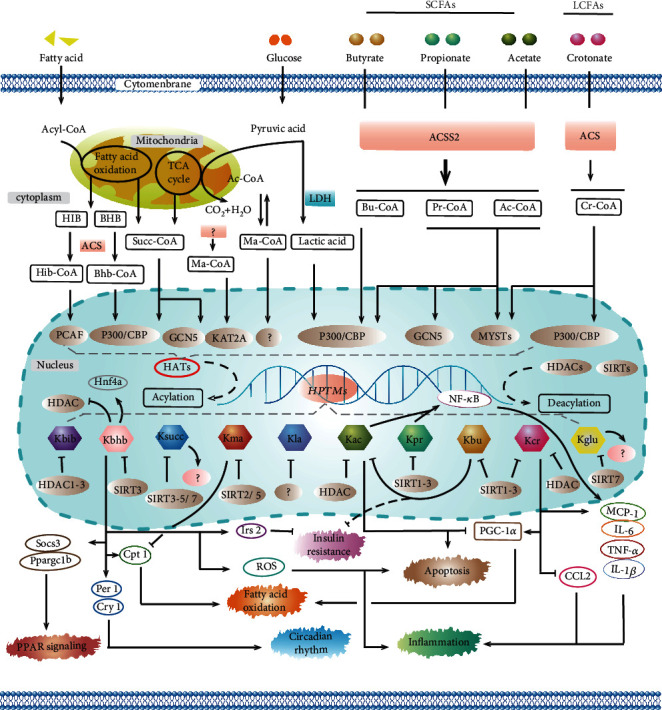
The summary of the processes and epigenetic mechanisms of novel HPTMs. Most HPTMs originate from acyl-CoA, which comes from the direct or indirect metabolism of glucose and fatty acids catalyzed by acyl-CoA synthetase (ACS). Acyl-CoA synthetase short-chain family member 2 (ACSS2) has been shown as a pivotal enzyme to promote HPTMs. HPTMs are modulated not only via HATs and HDACs but also via the sirtuin family. It has been verified that HPTMs participate in the regulation of insulin resistance, apoptosis, inflammation, fatty acid oxidation, circadian rhythm, PPAR signaling, and so on. The current prospects for novel HPTMs look attractive, and the related researches have obtained preliminary achievement. However, further studies are necessary to identify the novel epigenetic biomarkers in a cell type-specific manner and to develop novel therapeutic modalities.

Although the pathophysiological mechanisms have not been fully elucidated and the precise molecular mechanisms of these modifications are still not sufficiently, as summarized in Figure 4, HPTMs are indeed involved in the regulation of insulin resistance, apoptosis, inflammation, fatty acid oxidation, circadian rhythm, PPAR signaling, and other processes. Due to the absence of data, we mention little about Kglu and Khib in this review. Interestingly, a modification on histone H3 called serotonylation [75] is the first reported modification of a glutamine residue on a nonmethylated histone. This finding extends the function of serotonin in the epigenetic field and suggests other possible monoamine modifications. Approaches for identifying these modifications using LC-MS/MS or ChIP-on-chip will help expand the repertoire and understanding of the novel HPTMs. Evaluating epigenetic changes in freshly or archived obtained biological samples from patient cohorts may help identify significant biomarker signatures. In this regard, more sensitive and specific tests should be developed in clinical practice to detect the changes in modification markers in common diseases (e.g., atherosclerosis, stroke, and diabetic nephropathy). Second, to preserve or enhance the production of tissue-protective molecules, therapeutic interventions may be started earlier with novel HPTMs or supplements of different acyl-CoA forms. Target intervention at key points in upstream or downstream pathways may also lead to new therapeutics for disease and could potentially erase metabolic memory.

Unlike genetics, HPTMs are mostly reversible, generating infusive opportunities for therapeutic intervention via emerging approaches that enable locus-specific control of epigenetic marks. Based on this prospect, the biomarkers of epigenetics could promote precision medicine to optimally stratify patients for effective treatment. Thus, exploration of novel HPTMs may yield new insights into the pathogenesis of human disease and histone code that, in turn, can be translated into new cellular biomarkers and developed into exciting therapeutic modalities. We expect more progress in years to come towards a deeper and integrated understanding of these novel HPTMs that ultimately shape the epigenetic landscape.

## Figures and Tables

**Table 1:** The chemical structure, writers, erasers, and biological effect of novel HPTMs.

Structure	HPTMs	Sites	Writers	Erasers	Readers	Biological effect	Ref.
The hydrophobic acyl	Kac	H3: k4, k9, k14, k18, k27, k36, k37, k56, k64, k79, k115, k122 H4: k5, k8, k12, k16, k20, k31, k77, k79, k91 H2A: k5, k9, k13, k15, k36, k74, k95, k118, k127, k129 H2B: k5, k11, k12, k15, k16, k20, k23, k24, k43, k46, k57, k85, 108, k116, k120, k126	(1) P300/CBP (2) GCN5/PCAF (3) MYSTs (Moz, Ybf2, Sas2, and Tip60) (4) TAFII250, *α*TAT1, NCoA-1, CLOCK	(1) HDACs (2) SIRTs	(1) Bromodomain (2) DPF domain (3) YEATS domain	Transcription (+) NF-*κ*B↑ STAT3↑ P53↑, PAF53↑ MSCI (-)	[13] [31] [54]
	Kpr	H3: k23, k56 H4: k5, k8, k12, k16, k31, k44, k77, k79, k91 H2A: k95, k125 H2B: not available	(1) P300 (2) GCN5/PCAF (3) MYSTs (Tip60, MOF, MOZ, and HBO1)	SIRT1-3	(1) Bromodomain (2) YEATS domain	Transcription (+) TLR4↑ P53↑	[20] [31] [33] [36]
	Kbu	H3: k9, k14, k18, k23, k27, k79, k115, k122 H4: k5, k8, k12, k16, k31, k44, k77, k79, k91 H2A: k95, k125 H2B: k5, k11, k12, k15, k16, k59, k77, k91	P300/CBP	SIRT1-3	(1) Bromodomain (2) YEATS domain	Transcription (+) P53↑ MSCI (-)	[31] [39]
	Kcr	H3: k4, k9, k18, k23, k27, k56, k79, k122 H4: k5, k8, k12, k16, k59, k77, k91 H2A: k36, k95, k118, k119, k125 H2B: k5, k11, k12, k15, k16, k20, k23, k34, k108, k116	(1) P300/CBP (2) GCN5 (3) MYSTs (MOF)	(1) SIRT1-3 (2) HDAC1-3	(1) YEATS domain (Taf14, AF9, Yaf9, ENL, Sas5) (2) DPF domain (DPF2, MOZ) (3) CDYL	Transcription (+) P53↑ MSCI (-)	[31] [49–55]

The acidic acyl	Ksucc	H3: k14, k23, k27, k56, k79, k122 H4: k12, k31, k77, k79, k91 H2A: k9, k36, k95 H2B: k5, k34, k43, k46, k85, k108, k116, k120	(1) P300/CBP (2) GCN5	(1) SIRT5 (2) SIRT7	YEATS domain (GAS41)	Transcription (+) DNA repair (?)	[31] [47, 48]
	Kma	H3: k14, k18, k23, k56, k79, k122 H4: k8, k31, k77, k79 H2A: k95 H2B: k5, k34, k43, k108, k116, k120	Not available	(1) SIRT2 (2) SIRT5	Not available	Transcription (-) Cpt1↓ Energy metabolism (?)	[31] [40, 41] [43]
	Kglu	H3: k14, k18, k23, k27, k56, k79, k115, k122 H4: k5, k12, k31, k59, k77, k79, k91 H2A: k95, k99, k118, k1199, k125 H2B: k16, k34, k43, k108, k116, k120	KAT2A	Sirt7	Not available	DNA damage (?)	[23]

The polar acyl	Kbhb	H3: k4, k9, k14, k18, k23, k27, k56, k79, k122 H4: k5, k8, k12, k31, k77, k91 H2A: k5, k9, k13, k36, k95, k118, k119, k125 H2B: k5, k11, k16, k20, k34, k85, k108, k116, k120	P300	(1) SIRT3 (2) HDAC3	Not available	Gene expression (+) Per1↑ Cry1↑ Hnf4a↑ Irs2↑ Cpt1a↑ Socs3↑ Ppargc1b↑	[25] [31] [54]
	Khib	H3: k4, k9, k14, k18, k23, k27, k36, k56, k64, k79, k122 H4: k5, k8, k12, k16, k31, k44, k59, k77, k79, k91 H2A: k5, k9, k36, k74, k75, k95, k118 H2B: k5, k12, k20, k23, k34, k43, k46, k57, k85, k108, k116, k120	PCAF	HDAC1-3	Not available	Transcription (+) Meiotic genes ↑ Postmeiotic genes↑ MSCI (-)	[31] [24]
	Kla	H3: k9, k14, k18, k23, k27, k56, k79 H4: k5, k8, k12, k16, k31, k77, k91 H2A: k11, k13, k115 H2B: k5, k11, k15, k16, k20, k23, k43, k85, k108, k116, k120	P300	Not available	Not available	Transcription (+) Arg1↑ P53↑	[26]

P300/CBP: P300/CREB-binding protein; GCN5: GCN5-related N-acetyltransferase; MYSTs: MYST family protein; HDACs: histone deacetylases; SIRTs: sirtuin family; PCAF: P300/CBP-associated factor; MOZ: also known as KAT6A; MOF: also known as KAT8; Tip60: also known as KAT5; TLR4: Toll-like receptor 4; MSCI: meiotic sex chromosome inactivation; DPF: double PHD finger; DPF2: also known as BAF45d; CDYL: chromodomain Y-like protein; (+): activation; (-): inhibition; (?): unknown; ↑: upregulation; ↓: downregulation.

**Table 2:** The relationship between novel HPTMs and human diseases.

Diseases	Novel HPTMs	Biological sample or diseases model	Biological effect	Ref.
Cancer	Kbu	NB in vitro (SH-SY5Y cells)	Kac and Kbu levels are remarkably increased by SAHA treatment, playing an antitumor effect.	[57]
		ESCC in vitro (TE3, KYSE180)	Kbu has pretty low abundance compared to Kac in both of two tumor cell lines (TE3, KYSE180), which has a link to the recurrence-free survival in different ESCC stages.	[5]
	Kcr	Colon epithelial in vivo and HCT116 in vitro	Kcr (H3K18) is closely related to Hippo and cGMP-PKG tumor signaling pathway and linked to cell cycle progression, showing an increase in the S and G2-M phase over G1 arrested cells.	[58]
		HCC in vitro	Improved Kcr level inhibits hepatoma cell migration and decreases hepatoma cell proliferation, correlating with the TNM stage.	[59]

Neuropsychiatric disorders	Kcr	mPFC of C57BL/6 mice in vivo	Kcr, mediated by CDYL, regulates CSDS-induced stress-susceptible rodent microdefeat-induced social avoidance behaviors, anhedonia, and CSDS-induced depression-like behaviors.	[11]
	Kbhb	Dexamethasone-induced mice in vivo and cortical neurons in vitro	The Kbhb (H3K9) may activate the BDNF gene and ameliorate depression behaviors.	[12]

Metabolic diseases	Kbu	DIO mice in vivo	The levels of Kpr, Kbu, and Kmal are downregulated in the liver of the prediabetic DIO mice.	[9]
	Kbhb	HEK293 cells in vitro and STZ-induced T1DM mouse model in vivo	Kbhb marks vary in response to elevated BHB levels in vivo and in vitro, which is associated with the upregulated gene expression in starvation-related metabolic pathways (amino acid catabolism, circadian rhythm, redox balance, PPAR signaling, and oxidative phosphorylation).	[25]
	Kma	High glucose-treated mouse NE4C in vitro and diabetes-induced NTD mice in vivo	Kma level is increased in brain tissues from mice with diabetes-induced NTDs and in NE4C treated with high glucose.	[44]

Reproductive diseases	Kbu	Mouse spermatogenic cells in vitro	Kbu (H4 K5K8) exists and is enriched at the TSSs of genes, similar to Kac, directly stimulating gene transcriptional activity and participating in the regulation of genome reorganization during spermatogenesis.	[39]
	Kcr	Mouse spermatogenic cells vitro CDYL transgenic mice	Downregulation of Kcr leads to the reduction of male fertility with a decreased epididymal sperm count and sperm cell motility in CDYL transgenic mice.	[65]

Kidney diseases	Kcr	Proximal tubular cells in vitro and AKI mice in vivo	Kcr is increased during acute kidney injury in vivo and vitro, with PGC-1*α* and SIRT3 upregulated and CCL-2 downregulated.	[7]
		MHP with K.F. in clinical trials	Kcr decreased in MHP compared with healthy control.	[66]

AIDS	Kcr	J-Lat A1 cells and U1 cells in vitro	Kcr disrupts HIV latency following the induction of ACSS2 in vitro and ex vivo.	[74]

ESCC: esophageal squamous cell carcinoma; NB: neuroblastoma; SH-SY5Y cells: a cell line routinely used for studying neuroblastoma; SAHA: an HDAC inhibitor; HCT116: human colon carcinoma cells; HCC: hepatocellular carcinoma; CDYL: chromodomain Y-like protein; mPFC: medial prefrontal cortex; CSDS: chronic social defeat stress; BDNF: brain-derived neurotrophic factor; DIO mice: diet-induced obese mice by high-fat diet; TSSs: transcription initiation sites; PGC-1 *α*: peroxisome proliferator-activated receptor gamma coactivator-1*α*; TWEAK: tumor necrosis factor-like weak inducer of apoptosis; HEK293: human embryonic kidney 293 cells; MHP: maintenance hemodialysis patients; K.F.: kidney failure; J-Lat A1 cells: derived from Jurkat cells harboring HIV LTR-Tat-GFP gene; U1 cells: promonocytic cell line harboring HIV proviruses with defective Tat gene.
